# The fine line between performance improvement and device practicality

**DOI:** 10.1038/s41467-018-07733-6

**Published:** 2018-12-10

## Abstract

A quantitative improvement in the performance of a technology in the applied physical sciences, whether it be a solar cell with higher conversion efficiency or a detector with greater sensitivity, is an important stamp on progress which can strengthen an application. However, it is often unclear how performance improvements alone can enable real-life applications; an improvement in a particular performance metric doesn’t necessarily bring realization of that technology any closer. Conversely, new ideas that could open the door to new functionality aren’t necessarily accompanied by immediately impressive metrics. How can laboratory findings be effectively translated into technological advances that become useful in everyday life? While we support the publication of performance improvements, we also wish to encourage authors to look beyond performance metrics alone when reporting their technological improvements and think about the pathway to a practical technology.

There is a significant emphasis in the current research climate on making research impactful by linking it to an application. Certainly, advances in performance metrics—efficiency, sensitivity, speed, capacity, intensity and so on—may help to enable technologies, yet how exactly the technology is applied in practice for the envisioned application should also be explored. As editors we work hard to walk the fine line in assessing the relative importance between performance improvements and ideas which enable new or improved functionality in a technology, and we take into consideration the exact nature of the advance relative to the current state and maturity of the field. But with the definition of progress varying so dramatically from one community to the next, how should the journal assess these advances? To examine the discord between performance metrics and enabling technology from various community perspectives, we have commissioned a series of comments in a variety of fields across the applied physical sciences. We take several representative key devices which have well-defined performance metrics but underutilized potential in order to highlight the pathway towards more impactful applied research. It is our hope that this comment series will shed light on some of the directions in applied physical sciences research that should be explored and we, as a journal, are looking forward to seeing many of these developments realised.

It is our hope that this comment series will shed light on some of the directions in applied physical sciences research that should be explored and we, as a journal, are looking forward to seeing many of these developments realised.

While solar cells have achieved impressive power energy conversion efficiencies in thin-film modules in laboratories, scaling up the device area at a reasonable manufacturing cost is still a substantial challenge. Paul Meredith and Ardalan Armin in their comment (10.1038/s41467-018-05514-9) discuss a solution that relies on thicker solar cells and better designed electrodes to extract the current generated. They believe this solution may be broadly applied to many semiconducting materials, ranging from polymers to oxides. Besides scaling, operation stability is another major obstacle for large-scale development of high-performance and low-cost perovskite solar cells. Perovskites are an earth-abundant oxide that is efficient in absorbing visible light, but susceptible to degradation when exposed to heat, humidity, oxygen or light. Yang Yang and Jingbi You et al. (10.1038/s41467-018-07255-1) detail rational approaches to solve the stability issue. They also advocate the introduction of standardized protocols for characterization of device lifetime in order to make fair comparisons between stability test results.

Introducing new materials into an established technology can be a very slow process. Thin-film transistor technology—one of the essential elements of integrated electronic circuits—is currently dominated by amorphous silicon-based materials. Current research is focused on searching for alternative semiconducting materials to silicon that can also maintain high charge carrier mobility—a measurement of how fast electrons or holes carrying currents migrate. However, the emphasis on high charge carrier mobility alone as a performance metric may be misplaced, according to Thomas Anthopoulos and Alexandra Paterson (10.1038/s41467-018-07424-2) and in the connection of large arrays, the device architecture and the device-to-device variation become equally important as carrier mobility.

Similarly, photodetectors—electronic devices that convert light into electricity—which are an essential component in a vast number of optoelectronic devices and applications, mainly rely on mature, low-cost conventional semiconductor technologies benefitting from a high level of integration with CMOS electronics. Efforts are underway towards alternative materials for photodetector designs with unique features over their traditional counterparts. In this scenario, two-dimensional (2D) materials —single atom layer materials—could play an important role by providing functionalities that are not easily attainable with traditional semiconductors, owing to their ultra-low weight, flexibility and therefore suitability for seamless integration on a variety of substrates. In his comment, Gerasimos Konstantatos, (10.1038/s41467-018-07643-7) discusses the roadmap for 2D material photodetectors along with the key metrics for device progression.

Translating these technologies from the lab to the ‘real world’ of industry and commercial application is far from straightforward. Nowhere is this more apparent than for battery technology and research, where increasingly impressive-sounding figures of merit, such as specific capacity and cycle life, are reported on an almost-daily basis. But in many cases, important practical considerations continue to be overlooked, limiting the majority of the battery chemistries and designs from being commercially viable. Chengdu Liang and Xinping Ai et al. (10.1038/s41467-018-07599-8) address these issues and call for a reconciliation of the gap between academia and industry, proposing a set of guidelines for best practice when assessing battery performance to help provide a more realistic view of their potential for commercial use.

The development of plasmonic sensors—which use the interaction between electrons and light to achieve a sensitive detection capability—has the potential to provide cross-disciplinary impact. However, the practical implications of improving figures of merit, such as sensitivity, can be unclear. Sang-Hyun Oh and Hatice Altug (10.1038/s41467-018-06419-3) emphasize how nano-scale plasmonic sensors should extend beyond just improving sensitivity and aim also to offer previously unachievable functionalities. By pushing light confinement to the limit with nanometre-scale structures, single-molecule detection and optical trapping are possible. However, it is the combination of novel material systems, metamaterial structures and spectroscopy techniques that will enable the next generation optical biosensors to offer more functionality, such as dynamic conformation sensing and improved biological interfacing with ultrasensitive detection.

Recent research efforts in the field of neuromorphic technology—electronic devices that emulate the electronic behaviour of neural network in our brains for information processing—have focused on achieving ultra-high density and energy-efficient operation for next-generation applications such as artificial intelligence. Despite significant progress at the single-device level, to achieve sophisticated neural network functions, it is essential to wire together hundreds of thousands unit devices on microchips; this then creates numerous system-level technological challenges that could hinder industrial adoption. Themis Prodromakis et al. (10.1038/s41467-018-07565-4) discuss the related performance metrics for hardware engineering in light of manufacturing challenges that prevent neuromorphic technology from going mainstream, and present a roadmap that highlights viable approaches for accelerating the fundamental research that might lead to realize energy-efficient brain-inspired computing.

The editors at *Nature Communications* feel passionately about their involvement in communicating applied physics research to the scientific community. We hope that this series lays out our hopes for the research in these fields and, with the help of experts from the community, identifies the avenues for further development for impactful usable technologies. A synergy between academia and industry will be crucial in determining whether these emerging technologies will play a crucial role for next-generation devices. In this context, we feel certain that awareness within the research community of both technology- and manufacturing- readiness levels will be key to entering into existing markets or opening up new ones.

